# Freeform vs. Aspheric Spectacle Lenses: A Comprehensive Review of Optical Performance, Clinical Outcomes, and Patient Considerations

**DOI:** 10.7759/cureus.104008

**Published:** 2026-02-21

**Authors:** Konstantinos Ladopoulos, Evangelos Pateras, Georgios Ninos

**Affiliations:** 1 Biomedical Sciences, Sector of Optics and Optometry, Laboratory of Optical Metrology, University of West Attica, Athens, GRC; 2 Optics and Optometry, University of West Attica, Athens, GRC

**Keywords:** aspheric lenses, contrast sensitivity, freeform lenses, patient’s satisfaction, peripheral aberrations, progressive addition lenses, spectacle lens design, visual acuity

## Abstract

Spectacle lens design has evolved from spherical lenses to aspheric lenses (ASL) and freeform lenses (FFL) to improve peripheral optics and wearer comfort. In this review, we compare ASLs and FFLs across optical performance, visual function, patient-reported outcomes, and practical considerations. We synthesized evidence from more than 90 peer-reviewed studies, technical reports, and authoritative textbooks. Databases included PubMed, Scopus, Web of Science, and specialist optometry sources. Eligibility focused on comparative ASL vs. FFL studies and high-quality reports. Data were organized into themes (optical performance, visual function, subjective outcomes, and practical issues).

Both ASLs and FFLs outperform spherical lenses centrally; however, FFLs demonstrate consistently lower peripheral astigmatism and mean power error, wider usable fields of view (particularly in progressive addition lenses (PALs)), and better contrast sensitivity in mesopic/low-contrast conditions. Personalization of position-of-wear parameters (vertex distance, pantoscopic tilt, wrap) is key to FFL advantages. Adaptation appears faster, and patient satisfaction is higher with FFLs, especially in complex prescriptions and progressive designs. The ASLs are cost-effective for moderate prescriptions and standard fits, while FFLs represent the benchmark for personalized spectacle correction, particularly for high ametropia and PALs, provided there are accurate measurements and quality-controlled manufacturing.

## Introduction and background

Spectacle lenses are utilized to address refractive errors by deviating or bending light to focus on the retina. Conventional spherical lenses cause large amounts of off-axis visual aberration, more specifically, peripheral astigmatism and power error, resulting in a poor quality of peripheral vision [1,2]. Aspheric lenses (ASLs) were developed to help mitigate these issues by using a front surface other than a spherical shape (most often a conic shape or polynomial) that increasingly flattens towards the periphery, resulting in a reduction of lens thickness and its corresponding oblique astigmatism and mean power error compared to its spherical equivalent [3-5]. There is also the option of using freeform lenses (FFLs), sometimes referred to as 'digital,' 'individualized,' or 'optimized' lenses, representing the highest level of technology available today, both in single vision and progressive addition lenses (PALs).

**Figure 1:**
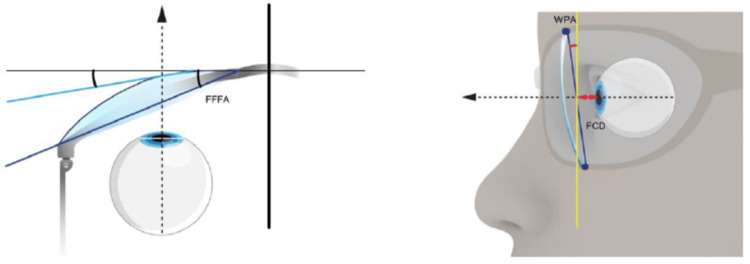
Individual position of wear FFFA: Frame face form angle, FCD: Frame-cornea distance, WPA: Wearer's pantoscopic angle Illustration sourced from *Assessment of Visual Quality Improvement as a Result of Spectacle Personalization* by Benyó et al, Life, 2023 (licensed under CC BY) [11].

Freeform lenses are manufactured using precision digital surfacing technology that operates based on more elaborate and complex mathematical algorithms. The digital surfacing process optimizes the entire back surface (or both surfaces) incrementally, or rather 'point by point,' based on the individual's prescription and variables unique to the lens wearer, such as their vertex distance, pantoscopic tilt, face form angle, frame shape, working distance (for PALs), and fitting height (Figure 1) [6-11]. Personalizing these dimensions aims to deliver superior optical performance across the entire lens, particularly in the periphery. This study presents a systematic comparison of key optical principles, performance factors, clinical advantages and disadvantages, and cost-benefit analyses of both ASLs and FFLs.

## Review

Methodology

Search Strategy

Searches in PubMed, Scopus, Web of Science, Cochrane Library, ResearchGate, and optometry databases (e.g., VisionCite) used keyword combinations including "freeform spectacle lenses," "digital lenses," "personalized lenses," "optimized lenses," "aspheric spectacle lenses," "spherical aberration," "peripheral aberrations," "visual acuity," "contrast sensitivity," "field of view," "patient satisfaction," "progressive addition lenses," and "ophthalmic optics." Boolean operators (AND, OR) tightened all searches. The literature search covered studies published from January 1974 through December 2025, with the final search performed on December 15, 2025. A total of 79 studies met the inclusion criteria and were incorporated into the qualitative synthesis.

Inclusion and Exclusion Criteria

Original, peer-reviewed articles (clinical trials, cohort studies, case-control studies), a review and/or meta-analysis, and technical reports from established and reputable institutions, as well as authoritative textbooks published in English, were included in the review. Emphasis was placed on studies that compared ASL and FFL performance (optical, visual, and subjective) directly. Animal studies and non-English articles, only abstracts without full text, theoretical modeling without testing, studies that only compared the spherical lens vs. ASL/FFL without ASL and FFL comparisons, and duplicates were excluded from the review.

Data Extraction and Synthesis

Information about lens designs, measurement procedures (including wavefront measurements, measures of visual acuity, questionnaires), study populations, primary outcomes (four broad outcomes: central visual/optical errors, peripheral visual/optical errors, measures of visual dysfunction like visual acuity or contrast sensitivity, measures of clear vision like distortion, and patient preference), and limitations was extracted. Results were synthesized within themes into four broad themes (optical performance, visual function/visual capacity, subjective outcomes, and issues relating to practicalities). The quality of the studies was assessed using appropriate tools, i.e., the Cochrane Risk of Bias tool [12] for randomized controlled trials (RCTs).

Optical principles and comparison of designs

Characteristics of ASLs

**Figure 2:**
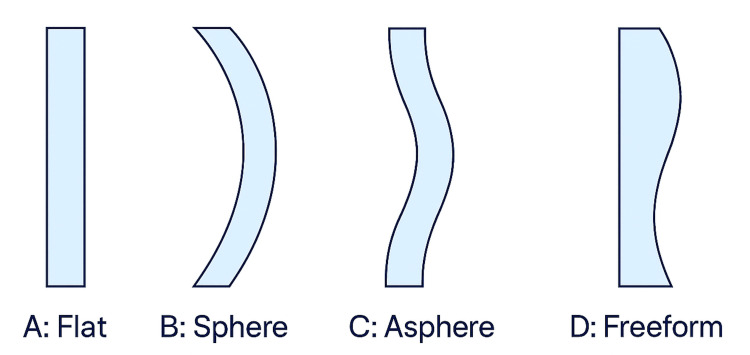
Representative conceptual surface profiles illustrating the evolution from flat and spherical designs to aspheric and freeform geometries While the illustration does not directly depict optical aberrations, such geometry transitions enable the reduction of spherical aberration, marginal astigmatism, and field curvature in modern ophthalmic and precision optics. Illustration created by the authors using Microsoft Copilot (Microsoft Corp., Redmond, WA, USA), a built‑in AI image generation tool.

**Figure 3:**
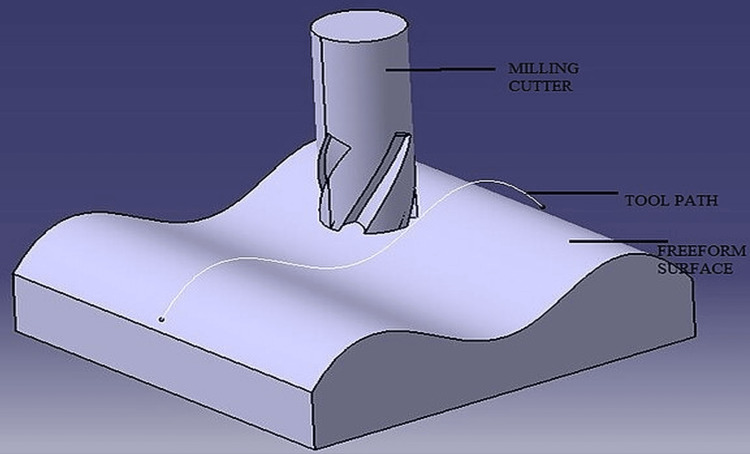
A 3D illustration of freeform surface milling representing the manufacturing capability required for the production of complex aspheric and freeform geometries Although the image shows fabrication rather than mathematical optimization, such processes enable the realization of surfaces like those optimized by Sun et al. [18]. Image credit: Abhishekmep (2014), via Wikimedia Commons, file: Freeform_surface_milling 2. JPG (licensed under CC BY).

Aspheric lenses use a front surface that is rotationally symmetric and can be defined by a conic constant (k-value) or a low-order polynomial [3,13,14]. The main aim is to diminish spherical aberration, marginal astigmatism (tangential and sagittal), and field curvature compared to a best-form spherical lens for a standard wearer position [4,15] (Figure 2). Designs are generally 'one-size-fits-most' for certain ranges of prescriptions and base curves. There are also other designs for ASLs. Katz tried to use two aspheric surfaces (bi-ASL), one at the anterior and one at the posterior surface of the lens, to have a better quality of vision [16]. Jalie also designed a variety of lenses (single vision, bifocal, and progressive) using a toroidal back surface [17]. In 2000, Sun et al. used one aspherical surface to optimize the aspherical surface coefficients for the same reason, i.e., improved quality of vision (Figure 3) [18].

Characteristics of FFLs

**Figure 4:**
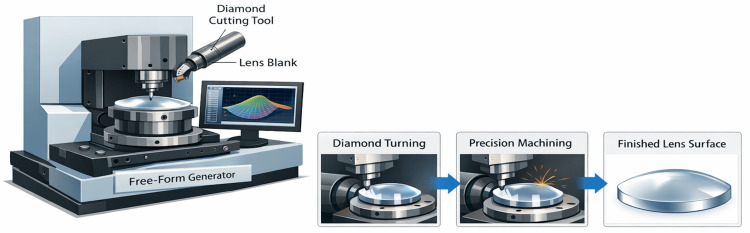
Overview of the FFL fabrication workflow FFL: Freeform lens; Illustration created by the authors using Microsoft Copilot, a built‑in AI image generation tool.

The FFLs utilize sophisticated algorithms (e.g., ray tracing, optimization merit functions) to compute a distinct non-rotationally symmetric back surface topography for each lens [6,19,20]. Specialized computer numerical control (CNC) generators and polishers, which have the ability to curve the lens in three dimensions (x, y, and z), produce these surfaces (Figure 4) [21]. The optimization accounts for eight parameters [7,22-24] to be taken into consideration, namely, (1) prescription (sphere, cylinder, axis, add power), (2) monocular pupillary distance (PD) and fitting height, (3) vertex distance, (4) pantoscopic tilt, (5) wrap angle (face form), (6) frame shape and curvature, (7) reading distance for PALs, and (8) wearer's visual priorities (e.g., distance, intermediate, near, or wider fields) [25]. As a result, FFLs can correctly target higher-order aberrations (HOAs) such as coma and trefoil, in addition to spherical aberration and astigmatism for the specific frame and individual fitting [26,27].

Optical performance comparison

Central Optical Quality

Both ASLs and FFLs markedly excel over spherical lenses at the center [28]. Studies typically have shown that best-corrected central visual acuity differs minimally between high-quality ASLs and FFLs under photopic conditions with easy prescriptions [29,30]. Notably, FFLs have slight benefits in reducing central wavefront error and HOAs when user-specific parameters deviate significantly from assumed standards [31,32]. Generally speaking, the key point for FFL benefits is the personalization of the parameters. Position of wear (pantoscopic angle, face form angle, vertex distance) is the most crucial of all.

Peripheral Optical Quality (Key Differentiator)

The FFLs show a large and persistent advantage in reducing oblique astigmatism and power error. Results from wavefront analysis and power mapping show that FFLs show significantly lower levels of peripheral astigmatism and mean power error than ASLs, especially at >20 to 30 degrees eccentricity and high plus or minus powers [33-36]. Sheedy et al. (2006) showed that FFLs can reduce peripheral astigmatism by as much as 50% when compared to standard ASLs [37]. Also, reduced HOAs from FFLs have been shown to be more effective in reducing peripheral coma, trefoil, and spherical aberration because of their ability to customize the correction for specific ray paths through the lens-eye system [26,38,39]. The effect of frame and fit is taken into account, and the advantages of FFLs in the periphery become more pronounced for larger frames, greater wrap angles, and more tilted pantoscopic angles than fits defined as 'standard' for ASLs are designed for [22,40]. Another major subject is distortion. Although both systems provide reduced distortion compared with spherical lenses, FFLs, particularly those with distortion management algorithms ('swim' and 'sway' control), exhibit objectively measurable improvements in perceived distortion, especially with high-minus lenses, and in PALs [41,42]. As a result, all these have wider fields and a more comfortable vision for most parts of the lenses. The benefits are more for PAL wearers or for people with very high ametropia. These types of lenses are available only in freeform design by some manufacturers.

Comparative visual performance and clinical outcomes

The FFLs consistently provide statistically significant and clinically meaningful advantages in peripheral visual acuity over ASLs, which is directly related to their better peripheral optical quality [35,43,44]. It is an advantage of the freeform technology, which can produce a single optical system (lens) with maximum performance. So, aberrations are corrected, and the field of view is expanded simultaneously in a freeform lens [45,46]. This is critical for peripheral awareness tasks (i.e., driving, sports). Under mesopic or low-contrast conditions, there are typically notable advantages for FFLs over ASLs related to superior control over HOA and straylight [47,48]. Data reveal FFLs generally outperform ASLs regarding contact lenses, particularly at intermediate and high spatial frequencies and low luminance [29,49,50]. Improved contrast sensitivity provides enhanced clarity in challenging light (dusk, fog, night driving). Studies consistently report a wider usable field of view, particularly in the near and intermediate zones of PALs, for FFLs compared to traditional and sometimes optimized aspheric PALs [51-53]. Reduced head movement and improved visual efficiency have also been observed. The FFLs are associated with shorter adaptation time, especially in first-time PAL wearers and those switching from a simpler design, and a greater frequency of immediately comfortable vision [54-56]. Reduced peripheral blur and distortion are likely significant contributors. In cases of high ametropia, the FFLs provide thinner edges for negative and flatter profiles for positive. The benefits of FFLs are the most pronounced in high myopia and hyperopia [57,58].

Freeform PALs represent the bulk of the premium PAL market. They offer wider corridors, larger reading zones, smoother progression, and also a significant reduction of peripheral blur ('swim effect') compared to traditional or aspherically-designed PAL design [59,21,60]. For example, Eiden et al. (2009) showed that patients prefer FFL PALs to prior designs in their clinic [61]. The FFLs provide superior stabilization of the cylinder axis, even in oblique meridians, and for non-orthogonal fittings, which may also stabilize vision [62,63]. Customized FFLs perform favorably for unique occupational design (e.g., large intermediate/near zone, and/or decided task zones, etc.) [25,64]. With FFLs, subjective patient satisfaction and preference are increased. Controlled studies and surveys report higher subjective satisfaction, preference, and perceived visual quality with FFLs compared to ASLs [65-67]. They present wider and clearer fields of vision [53,68]. Also, there is a faster adaptation time, particularly with PALs [54] and superior low-light/night driving [47,69]. There is less distortion and movement perception ('swim') sensation [41,70], and a general feeling of 'crisper' or 'more natural' vision [71]. In FFLs, we can have different curvatures at the superior than the inferior part of the lens. At the inferior parts of PALs, we can create surfaces with a bigger front curvature than the superior parts.

**Figure 5:**
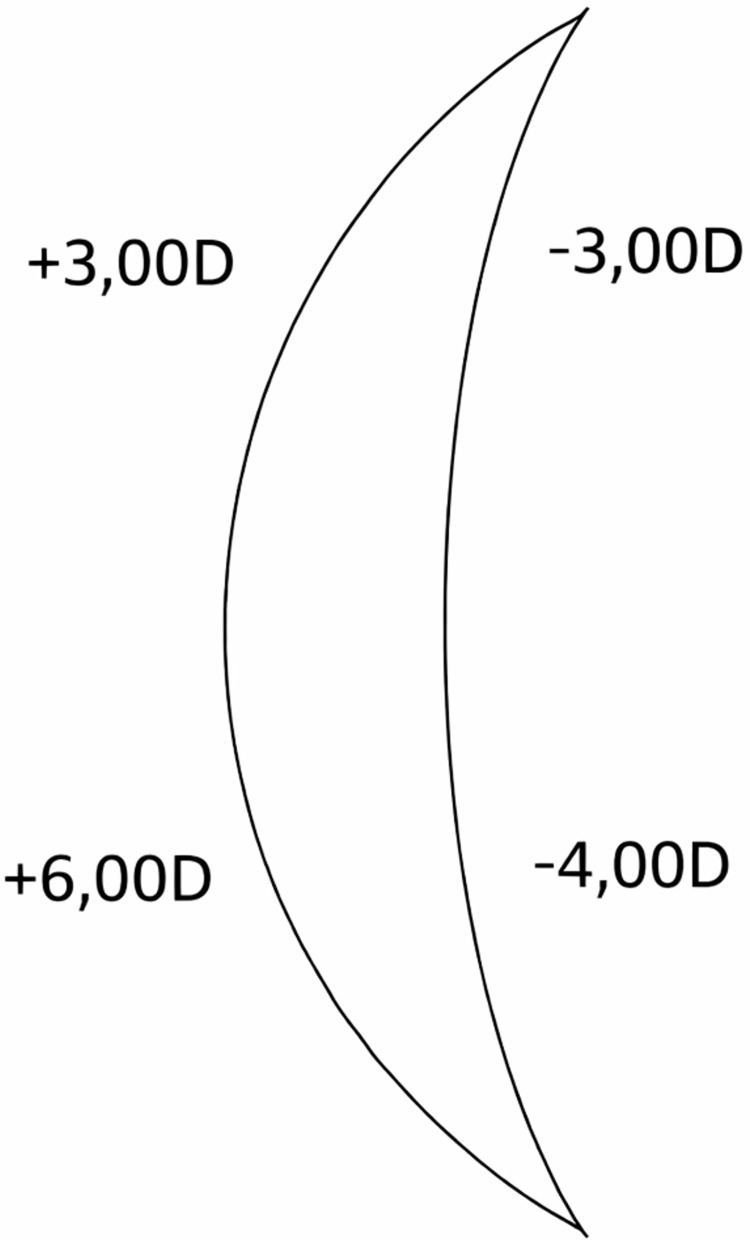
The curvatures of anterior and posterior surfaces of a freeform PAL (plano addition (ADD) 2.00) PAL: Progressive addition lens; Illustration created by the authors using Microsoft Copilot, a built‑in AI image generation tool.

We already know that the angular magnification of a lens (M) is given by the type $M = \frac{1}{1 - \frac{t}{n} P_1} \times \frac{1}{1 - d P_v}$, where 't' is the thickness of the lens, 'n' the refractive index, 'd' the vertex distance, 'P1' the power of the front surface of the lens, and 'Pv' the back-vertex power. It is obvious that, as P1 increases, the first term (shape factor) of the above equation grows bigger. So, for bigger power front surfaces, we have bigger magnification. This is very critical for near vision because we have better visual quality with such a lens (Figure 5).

In terms of practical considerations and limitations, the cost and value of FFLs compared to ASLs typically include a significant price premium due to the complexities associated with the design, manufacturing, and measurement process [72]. Aspheric lenses provide excellent value where prescriptions are moderate, and the fit is more standard in frame style. The value of FFLs is most evident in high-strength prescriptions or complex corrections (high astigmatism, strong adds), non-standard fits, large wrapped frames, patients with high visual demands, or sensitivity to aberrations/distortion [73]. With FFLs, the measurement requirements must be more accurate. The loss of benefit associated with an FFL can be attributed exclusively to measurement error in one or more of the fitting parameters (monocular PD, fitting height, vertex distance, pantoscopic tilt, face form) [74], and accuracy in these measurements is key to realizing the maximum benefits of FFLs [75]. There is also evidence that inaccurate measurements can eliminate the benefit associated with an FFL and possibly degrade performance to that of a well-fitted ASL [76]. For manufacturing, tolerance, and quality control, FFL requires highly precise digital surfacing equipment. Variabilities in manufacturing tolerances could potentially impact the optical performance of the prescription delivered to the patient [77,78]. The FFLs are readily available commercially; however, they still require specific lab machinery and expertise to produce. Basic ASLs are ubiquitous.

Discussion and clinical implications

Table 1 shows a brief, overall, comparative summary outcome concerning ASL and FFL characteristics. The evidence is quite clear that FFLs outperform ASLs when it comes to reducing peripheral aberrations, increasing contrast sensitivity, expanding the field of clear vision, and improving subjective visual quality and comfort. This is due to the very nature of freeform technology allowing for specific customization of the lens surface according to the individual's prescription, facial anatomy, and frame selection. The ASLs are still a reasonably acceptable, cost-effective solution for how to moderate spherical aberration. They are worn in standard frames without excessive angle of tilt or wrap. When the budget may be a consideration, the demand for perfection in single vision distance correction is less for peripheral use than for more complex lenses. The FFLs are preferable for higher prescriptions (hyperopia > +4.00, myopia < -4.00, astigmatism > 2.00). The PALs are prescribed for standard frames with substantial wrap, large frames, or non-standard fit (e.g., high pantoscopic tilt). Also, FFLs can be used for high-demand visual use (driving, night driving, computer use, sports). Custom FFLs are essential for occupational lens designs. The performance difference between the FFLs and ASLs is optimized with FFLs when the refraction demands it, and a full set of measurements is taken (monocular PD, fitting height, vertex, tilt, wrap) [77,78]. If the measurements are wrong, the premium FFL will outperform an ASL that has been properly fitted [79,80].

**Table 1:** Comparison of ASLs and FFLs ASLs: Aspheric lenses, FFLs: Freeform lenses, PALs: Progressive addition lenses

Feature	ASLs	FFLs
Optical performance	Improved over spherical lenses; limited personalization	Superior performance; fully personalized
Peripheral aberrations	Reduced compared to spherical lenses	Significantly minimized; up to 50% reduction
Contrast sensitivity	Moderate improvement	Enhanced at all spatial frequencies
Adaptation time	Short for single vision; longer for PALs	Very short; smoother adaptation for PALs
Cost	Economical for standard prescriptions	Higher or premium pricing
Customization	Limited to design parameters	Full customization based on the wearer’s measurements

## Conclusions

The ASLs represent a significant performance upgrade over spherical lenses, enabling a reduction in central thickness and improvements in peripheral optics for standard-fit lenses. The FFLs mark a revolutionary stage in lens technology, enabling custom levels of personalization and optimization that were previously not achievable in spectacle correction. While ASLs provide excellent value for many standard prescriptions, FFLs will outperform ASLs and provide wider fields of clear vision, greater contrast sensitivity, higher subjective satisfaction, and the fastest adaptation in prescriptions, especially in complex prescriptions (hyperopia or myopia greater than 4D and astigmatism greater than 2D, prism prescriptions, etc.), non-standard fit prescriptions (large wraps, high pantoscopic tilt, reduced/extended vertex distance), progressive lenses, or patients with complex or high demands on their visual system. The ultimate decision between an ASL and FFL still comes down to the detailed assessment of the patient's prescription, frame selection, visual needs, lifestyle, and budget, as well as the willingness of the practitioner to take the necessary time to take detailed measurements for optimization. The FFLs are now the current benchmark in lens technology to obtain the highest level of visual performance and comfort for eyeglasses.
